# LAPAROSCOPIC RIGHT AND LEFT COLECTOMY: WHICH PROVIDES BETTER POSTOPERATIVE RESULTS FOR ONCOLOGY PATIENTS?

**DOI:** 10.1590/0102-672020230074e1792

**Published:** 2024-02-05

**Authors:** Rodrigo Ambar Pinto, Diego Fernandes Maia Soares, Lucas Gerbasi, Caio Sérgio Rizkallah Nahas, Carlos Frederico Sparapan Marques, Leonardo Alfonso Bustamante-Lopes, Mariane Gouvea Monteiro de Camargo, Sérgio Carlos Nahas

**Affiliations:** 1 Universidade de São Paulo, Faculty of Medicine, Department of Gastroenterology, Coloproctology Unit - São Paulo (SP), Brazil.

**Keywords:** Colorectal Surgery, Colectomy, Intestinal Neoplasms, Morbidity, Cirurgia Colorretal, Colectomia, Neoplasias Intestinais, Morbidade

## Abstract

**BACKGROUND:**

The laparoscopic approach considerably reduced the morbidity of colorectal surgery when compared to the open approach. Among its benefits, we can highlight less intraoperative bleeding, early oral intake, lower rates of surgical site infection, incisional hernia, and postoperative pain, and earlier hospital discharge.

**AIMS:**

To compare the perioperative morbidity of right versus left colectomy for cancer and the quality of laparoscopic oncologic resection.

**METHODS:**

Retrospective analysis of patients submitted to laparoscopic right and left colctomy between 2006 and 2016. Postoperative complications were classified using the Clavien-Dindo scale, 30 days after surgery.

**RESULTS:**

A total of 293 patients were analyzed, 97 right colectomies (33.1%) and 196 left colectomies (66.9%). The averageage was 62.8 years. The groups were comparable in terms of age, comorbidities, body mass index, and the American Society of Anesthesiology (ASA) classification. Preoperative transfusion was higher in the right colectomy group (5.1% versus 0.4%, p=0.004, p<0.05). Overall, 233 patients (79.5%) had no complications. Complications found were grade I and II in 62 patients (21.1%) and grade III to V in 37 (12.6%). Twenty-three patients (7.8%) underwent reoperation. The comparison between left and right colectomy was not statistically different for operative time, conversion, reoperation, severe postoperative complications, and length of stay. The anastomotic leak rate was comparable in both groups(5.6% versus 2.1%, p=0.232, p>0.05). The oncological results were similar in both surgeries. In multiple logistic regression, ASA statistically influenced the worst results (≥ III; p=0.029, p<0.05).

**CONCLUSIONS:**

The surgical and oncological results of laparoscopic right and left colectomies are similar, making this the preferred approach for both procedures.

## RESUMO

**RACIONAL:**

A abordagem laparoscópica reduziu consideravelmente a morbidade da cirurgia colorretal quando comparada à abordagem aberta. Entre seus benefícios podemos destacar o menor sangramento intraoperatório, ingestão oral precoce, menor índice de infecção de incisão cirúrgica e hérnia incisional, menor índice de dor pós-operatória e alta hospitalar mais precoce.

**OBJETIVOS:**

Comparar a morbidade perioperatória da colectomia direita versus esquerda para câncer e a qualidade da ressecção oncológica laparoscópica.

**MÉTODOS:**

Análise retrospectiva de pacientes submetidos à olectomia laparoscópica direit e esquerda entre 2006 e 2016. As complicações pós-operatórias foram classificadas pela escala Clavien-Dindo, 30 dias após a cirurgia.

**RESULTADOS:**

Um total de 293 pacientes foram analisados, 97 casos de colectomia direita (33.1%) e 196 de esquerda (66.9%). A idade média foi de 62,8 anos. Os grupos foram comparáveis em termos de idade, comorbidades, índice de massa corporal e classificação da Sociedade Americana de Anestesiologia (ASA). A transfusão pré-operatória foi maior no grupo da colectomia direita (5,1% versus 0,4%, p=0,004, p<0,05). No geral, 233 pacientes (79.5%) não apresentaram complicações. As complicações encontradas foram graus I e II em 62 pacientes (21,1%), egraus III a V em 37 (12,6%). Vinte e três pacientes (7,8%) foram reoperados. A comparação entre a colectomia laparoscópica esquerda e direita não foi estatisticamente diferente para tempo operatório, conversão, reoperação, complicações pós-operatórias graves e tempo de internação. A taxa de fístula anastomótica foi comparável em ambos os grupos (5,6% versus 2,1%, p=0,232, p>0,05). Os resultados oncológicos foram semelhantes nas duas cirurgias. Na regressão logística múltipla, a ASA influenciou estatisticamente os piores resultados (≥ III; p=0,029, p<0,05).

**CONCLUSÕES:**

Os resultados cirúrgicos e oncológicos das colectomias laparoscópicas direita e esquerda são semelhantes, tornando esta a abordagem preferida para ambos os procedimentos.

## INTRODUCTION

The laparoscopic approach considerably reduced the morbidity of colorectal surgery when compared to open surgery. Among its benefits, we can highlight less intraoperative bleeding, early refeeding, lower rates of surgical site infections, incisional hernias, and postoperative pain, and earlier hospital discharge^5,12,16^. It is also well established that the videolaparoscopic approach has oncological equivalence compared to the open approach^1,5,7,13^.

Left colectomy is technically considered more difficult, as it requires colorectal or colo-colic anastomosis, which has a higher incidence of dehiscence, surgical site infection, and longer hospital stay than the right colectomy, in which ileocolic anastomosis is performed^7,15,17,18^. However, a few studies have compared the two procedures in relation to intraoperative complications and postoperative results^7,11,13,15^, and the literature becomes even scarcer if only the laparoscopic approach is considered.

The objectives of the present study are to compare preoperative characteristics, intraoperative complications, and postoperative results, including the quality of oncological resection, and evaluate the surgical specimen, between patients undergoing right colectomy and left colectomy via laparoscopy.

## METHODS

The research was approved by the Institution's Medical Ethics Committee (number 090819), and patient information was obtained through retrospective analysis of a prospective database of patients undergoing elective right or left laparoscopic colectomy for the management of colon cancer at the University Hospital of the Faculty of Medicine of the Universidade de São Paulo from 2006 to 2016. Patients who underwent right or left partial colectomy with lymphadenectomy and aged 18 years and older were included. Patients undergoing total colectomy, resections that included the rectum, transverse resections, multivisceral resections, and those who did not have an immediate reconstruction of intestinal transit were excluded.

The groups were compared according to characteristics: age, comorbidities, body mass index, American Society of Anesthesiology (ASA) classification, operative time, intraoperative complications, length of stay, perioperative blood transfusion, preoperative hemoglobin — operative and postoperative, number of lymph nodes resected, oncological staging (TNM — Tumor Lymph node Metastases and AJCC — American Joint Committee on Cancer), postoperative complications, reoperations, and mortality.

Intraoperative complications were defined as bleeding, ureteral injury, intestinal perforation, or any other complication that deviated from the normal resection procedure, requiring immediate resolution during surgery. Preoperative hemoglobin measurement was defined as hemoglobin immediately before the procedure and postoperative hemoglobin was defined as that measured on the first day after surgery.

Among the postoperative complications occurring within 30 days after surgery, the following were collected: clinical complications, ileus, surgical site infection, anastomotic dehiscence, bleeding, and mortality. Complications were classified using the Clavien-Dindo classification^4^, with a grade III or higher being considered a serious complication. The quality of oncological resection was assessed by the mean number of lymph nodes and surgical margins.

Neoplasia of the left colon was defined as those located from the splenic flexure to the recto-sigmoid junction. During all resections of the left colon, the splenic flexure was completely released and intra-abdominal mechanical anastomosis was performed by double stapling.

Right colon neoplasia included lesions located from the ileo-cecal valve to the hepatic flexure. Resections of the right colon were followed by ileo-transverse anastomosis, using two techniques in this case series: mechanical isoperistaltic intra-abdominal anastomosis followed by closing the stapler passage hole manually and removing the specimen through a Pfannenstiel incision; or extracorporeal anastomosis, with an incision in the umbilical region for antiperistatical mechanical side-to-side anastomosis and removal of the specimen. All surgeries, both right and left colectomies, were performed by surgical residents in training, under the supervision of an assistant surgeon specialized in colorectal laparoscopy.

### Statistical analysis

For statistical analysis, quantitative characteristics were described through summary measurements (mean, standard deviation, median, minimum, and maximum values) and compared using Student's *t*-test or Mann-Whitney *U* test according to the normality distribution of data evaluated by the Kolmogorov-Smirnov test^9^. Qualitative characteristics were described using absolute and relative frequencies and the existence of an association was verified using chi-square tests or exact tests (Fisher or likelihood ratio).

Quantitative measurements were compared between patients with Clavien <3 and ≥3 using Student's *t*-tests and the association of qualitative characteristics with this outcome was verified using chi-square or exact tests. The odds ratio of each variable with the Clavien outcome was estimated with the respective 95% confidence intervals using bivariate logistic regression^8,9^. The multiple logistic regression model was estimated to explain Clavien >3 by selecting the variables that presented a descriptive level lower than 0.2 in the bivariate analyses.

Statistical Package for Social Sciences (IBM-SPSS) for Windows version 20.0^®^ software was used for the analyses and Microsoft Excel 2003^®^ software was used to tabulate the data. The tests were performed at a significance level of 5% (p<0.05).

## RESULTS

A total of 293 patients were included in the study and analyzed. Of these patients, 97 (33.1%) underwent right colectomy (RC) and 196 (66,9%) left colectomy (LC). Patients undergoing RC were older, with a higher rate of comorbidities and greater severity of comorbidities. Preoperative hemoglobin was lower in this group, which led to a higher preoperative transfusion rate (Table 1).

**Table 1:** Baseline data.

Variables		RC (n = 97)	LC (n = 196)	Total (n = 293)	p-value
Age (years)		67.7±11.6	61.3±11.4	63.4±11.8	<0.001
Gender (%)					
	Male	33 (34.0)	80 (40.8)	113 (38.6)	0.261
	Female	64 (66.0)	116 (59.2)	180 (61.4)	
Comorbidity (%)		78 (81.3)	131 (67.5)	209 (72.1)	0.014
BMI (kg/m^2^)		25.9±4.6	26.4±4.7	26.2±4.6	0.360
ASA (%)					
	I	9 (9.4)	43 (22.1)		
	II	67 (69.8)	129 (66.2)		0.010
	III	20 (20.8)	22 (11.3)		
	IV	0 (0)	1 (0.5)		
Preop. Hb (g/dl)		11.7±2.1	12.7±1.7	12.4±1.9	<0.001
Preop. transfusion (%)		7 (7.2)	4 (2.0)	11 (3.8)	0.045
Previous surgery (%)		38 (39.2)	66 (33.7)	104 (35.5)	0.354

RC: right colectomy; LC: left colectomy; n: number; BMI: body mass index; ASA: American Society of Anesthesiologists; Preop.: preoperative; Hb: hemoglobin.

Surgical time was longer in LC 228.8±62.1 min vs. RC 195±54.1 min (p<0.001). There was no statistically significant difference between the groups when compared in terms of conversion rate p=0.98 (6.2% RC vs. 6.1% LC). The main reason for conversion was the presence of intestinal adhesions due to previous abdominal surgeries, with 4 (4.1%) cases in the RC group and 5 (2.5%) in the LC group. The rate of intraoperative complications was 3.7% (11 cases), with no difference between RC and LC (3.1% vs. 4.1% respectively, p>0.99). Intraoperative complications were: ureter injury at 0.6% (2 cases), bleeding at 2.0% (6 cases), and small intestine injury at 1.0% (3 cases), as described in Table 2.

**Table 2:** Intraoperative results.

Variables	RC (n = 97)	LC (n = 196)	Total (n=293)	p-value
Conversion (%)	6 (6.2)	12 (6.1)	18 (6.1)	0.983
Intraop. complications (%)	3 (3.1)	8 (4.1)	11 (3.8)	>0.999
Operative time (min)	195.0±54.1	228.8±62.1	217.7±61.6	<0.001
Postop. Hb (g/dl)	10.6±1.8	11.2±1.6	11±1.7	0.004
Postop. transfusion (%)	7 (7.2)	8 (4.1)	15 (5.1)	0.268

RC: right colectomy; LC: left colectomy; n: number; Intraop.: intraoperative; min.: minutes; Postop.: postoperative; Hb: hemoglobin.

The overall complication rate was 34% in RC and 23.5% in LC (Table 3). Clinical complications were more frequent in RC (19.6% vs. 7.1%; p=0.002, p<0.05). There was no difference between the groups regarding Clavien-Dindo classification of postoperative complications and 30-day mortality.

**Table 3:** Intraoperative complications.

Variables	RC (n=97)	RC (n = 196)	Total (n = 293)	p-value
Morbidity (%)	33 (34)	46 (23.5)	79 (27)	0.055
Length of stay (days)	8.2±6.7	7.6±3.9	7.5±5	0.932
Anastomotic leak (%)	2 (2.1)	11 (5.6)	13 (4.4)	0.232
Reoperation (%)	5 (5.2)	10 (5.1)	15 (5.1)	>0.990
Severe complication (%) (Clavien > III)	10 (10.3)	14 (7.1)	24 (8.2)	0.352
Mortality (%)	3 (3.1)	2 (1.0)	5 (1.7)	0.336

RC: right colectomy; LC: left colectomy.

The oncological results, evaluating the number of lymph nodes resected, showed no statistical difference between the two groups analyzed, with an average of 19.4 lymph nodes dissected in RC and 18.5 in LC. However, neoplasms located in the right colon were more advanced when evaluating the T parameter (p=0.048, p<0.05; Table 4). However, it did not cause a statistically significant difference in the final pathological staging of the patients (p=0.145, p>0.05; Table 5).

**Table 4:** Oncologic resection evaluation by T stage.

T stage (%)	RC (n = 97)	LC (n = 196)	Total (n = 293)
Tis	12 (12.4)	23 (11.7)	35 (11.9)
I	2 (2.1)	21 (10.7)	23 (7.8)
II	13 (13.4)	30 (15.3)	43 (14.7)
III	62 (63.9)	110 (56.1)	172 (58.7)
IVa	8 (8.2)	10 (5.1)	18 (6.1)

RC: right colectomy; LC: left colectomy; T: tumor wall invasion; Tis: mucosal tumor.

**Table 5:** Oncologic resection evaluation by pathologic final stage.

Final stage (%)	RC (n = 97)	LC (n = 196)	Total (n = 293)
0	12 (12.4)	24 (12.2)	36 (12.3)
I	9 (9.3)	42 (21.4)	51 (17.4)
IIA	28 (28.3)	63 (34.1)	91 (31.1)
IIB	4 (4.1)	7 (3.6)	11 (3.8)
IIIA	5 (5.2)	8 (4.1)	13 (4.4)
IIIB	32 (33.0)	40 (20.4)	72 (24.6)
IIIC	3 (3.1)	4 (2.0)	7 (2.4)
IVA	4 (4.1)	8 (4.1)	12 (4.1)

RC: right colectomy; LC: left colectomy.

Bivariate logistic regression showed that there were worse complications, according to the Clavien-Dindo classification^4^, in patients with higher ASA (p=0.035, p<0.05), patients undergoing blood transfusion (p=0.019), and those who presented intraoperative small intestine injury (p=0.006, p<0.05). In the multiple logistic regression, the parameter injury to the small intestine was not included since 100% had a worse Clavien-Dindo classification^4^, in addition to being an outcome.

Table 6 shows that together only ASA statistically influenced, independently, the worst Clavien-Dindo outcome (≥3; p=0.029, p<0.05), with each increase in ASA the chance of a worse outcome was 2.79 times greater as compared to the previous ASA.

**Table 6:** Multiple logistic model results to explain the occurrence of Clavien-Dindo classification ≥ 3.

Variable	OR	95%CI		p-value
		Inferior	Superior	
Gender (male)	1.37	0.51	3.70	0.533
Age (years)	0.97	0.93	1.01	0.186
BMI (kg/m^2^)	0.90	0.80	1.02	0.092
ASA	2.79	1.11	7.01	0.029
Palliative	2.32	0.53	10.26	0.267
Transfusion	2.18	0.36	13.13	0.396
LC	0.94	0.33	2.70	0.910

BMI: body mass index; ASA: American Society of Anesthesiologists; LC: left colectomy; OR: odds ratio; CI: confidence interval.

## DISCUSSION

RC is, in general, seen as less morbid by most surgeons, being the surgery of choice to begin the colorectal surgery learning curve. This is probably because the rate of anastomotic leakage is lower than that of LC, causing less anxiety for the surgeon.

In the present study, the rate of postoperative complications between the two laparoscopic resections was similar, showing that both procedures are equally safe. Even though patients undergoing RC have a greater number of comorbidities, resulting in a worse clinical condition and higher ASA, older age and more advanced clinical staging at diagnosis, postoperative outcomes were similar.

A positive point of this study was limiting the analysis to the laparoscopic approach to colon cancer, making the comparison of technical aspects and results more homogeneous and plausible than in other series that included multiple etiologies and different procedures in the same analysis.

Studies comparing right to left colectomy are the minority in the literature. Of the most recent literature found, the first four studies primarily evaluated the open surgery and the last three evaluated the laparoscopic (Table 7). Of the four studies that compared RC to open LC^1,6,9,11^, three were population-based with a large number of patients. Only Kwaan et al.^10^ included 42% of laparoscopic colorectal surgeries. Of these, three studies^1,10,13^ included only cancers and the other mixed with benign diseases^7^. Pre-existing comorbidities, described in three studies, were more prevalent in RC. Postoperative morbidity, also described in two of the four studies, varied. They were more frequent in LC according to Hinojosa et al.^7^ (22.9% vs. 25.3%, respectively for RC and LC) and in RC according to Masoomi et al.^13^ (28.43% vs. 23.75%, respectively for RC and LC).

**Table 7:** Literature studies results comparing right to left colectomy.

	Hinojosa et al.^7^	Benedix et al.^1^	Masoomi et al.^13^	Kwaan et al.^10^	Turrado-Rodriguez et al.^16^	Nfonsam et al.^14^	Campana et al.^2^	This research, 2023
Patients	27,483	17,641	50,799	4,875	1,000	2,512	547	293
RC/LC	12,9/14,5%	8,927/9,344	32,277/18,522	2,222/2,653	499/501	1,256/1,256	292/255	97/196
Approach	Open (100%)	Open (100%)	Open (90.4%)	Open (58.0%)	Laparoscopic	Laparoscopic	Laparoscopic	Laparoscopic
Diseases included	Benign and malignant	Malignant	Malignant	Malignant	Benign and malignant	Malignant	Malignant	Malignant
Comorbidities	–	>RC	>RC	>RC	equivalent	–	>RC	> RC
Operative time	–	–	–	>LC	>LC	–	>LC	> LC
Intraoperative complications	–	>LC	>LC	–	>RC	Equivalent	Equivalent	Equivalent
Postoperative morbidity	>LC	Equivalent	>RC	Equivalent	>LC	>LC	>RC	Tendency >RC
Length of stay	>LC	–	Equivalent	>RC	Equivalent	>LC	>RC	Equivalent
Mortality	Equivalent	>RC	Equivalent	Equivalent	Equivalent	Equivalent	Equivalent	Equivalent

The most recent study that analyzed the laparoscopic approach was retrospective and presented a more limited number of patients; it not only evaluated patients with cancer but also included benign diseases in the cases analyzed^3,14,16^. As in the present study, according to Campana et al., preexisting comorbidities were more prevalent in the RC group^2^.

Operative time, described in other studies, and also in the present study, was longer in LC. In our series, all patients underwent mobilization of the splenic flexure, which could explain the longer operative time, as also mentioned by Campana et al.^2^. Mobilization of the splenic flexure can increase operative time, on average, by 35 minutes^3^.

Intraoperative complications were similar in two other studies, as well as in the present study, but they were more frequent in RC, according to Turrado-Rodrigues et al.^16^.

Only the present study and another one in the verified literature classified the severity of postoperative complications according to Clavien-Dindo^4^, also presenting no statistically significant differences, comparing both right and left resections for the treatment of colon cancer^16^.

Regarding postoperative morbidity, Campana et al.^2^ reported a higher rate of 20.5% in RC as compared to 13.3% in LC. It is worth mentioning that the Campana et al.^2^ case series is comparable to the present study, including only cancer patients undergoing laparoscopic surgery. On the other hand, Turrado-Rodriguez et al.^16^, had greater postoperative complications in LC (30% vs. 19%; p<0.0001), analyzing laparoscopic colectomies in benign diseases, with 14.2% of diverticular disease, which generally presents greater post-operative morbidity, justifying the worse immediate outcomes found in LC^14^.

Nfonsam et al.^14^ also recorded higher rates of surgical wound complications related to LC, and higher rates of reoperations due to surgical wound infection (56% vs. 35%; p = 0.010). The authors did not evaluate any other complications regarding surgery for colon cancer in the series described above. In the present study, there was no statistically significant difference in overall morbidity after surgery, but it was 34% in RC and 23.5% in LC (p=0.055, p>0.05), having a tendency towards greater complications in RC.

Postoperative clinical complications were more frequently associated to RC in the present study (19.6% vs. 7.1%; p=0.002, p<0.05), which may be associated with the greater morbidity of these patients before surgery and the greater risk of more severe postoperative complications, with Clavien-Dindo ≥3, in patients with higher ASA (OR 2.79).

In the series involving a majority of conventional surgeries, three studies evaluated the length of hospital stay. Hinojosa et al.^7^ observed a longer hospital stay in LC (6.2 vs. 7.2 days). Kawaan et al.^10^, in turn, found a direct relationship between postoperative complications and longer hospital stay.

Analyzing purely laparoscopic cases, the average length of stay after surgery was longer in LC according to Nfonsam et al.^14^ (10.5±4 vs. 7.1±1.3 days; p=0.02, p<0.05), while Campana et al.^2^ observed the opposite (3.9 vs. 4.7 days; p=0.003, p<0.05), and Turrado-Rodrigues et al.^16^ found no statistical differences in surgeries performed on the right or left sides, both in benign and malignant diseases. The length of stay was directly related to postoperative morbidity, mainly concerning prolonged ileus or surgical site infection, and a higher rate of reoperations.

Some studies that also compared RC and LC obtained similar results regarding older age, comorbidities rate, and more advanced clinical staging in patients undergoing RC. Most studies showed a large variation in perioperative morbidity and similar mortality between groups^7-9,14-18^. Only one study showed higher mortality in the RC group^1^; however, we must emphasize that the patients included were not operated via laparoscopy.

The overall mortality rate in our study was 1.7%, considered high as compared to other studies in the literature with laparoscopic resections, which ranged from 0.5 to 1.5%^3,14,16^. However, it can be justified by the patients' delay in searching for a diagnosis and arriving at specialized medical services for treatment in a worse general condition^11^. Besides, the mortality rate for isolated RC of 3.1% is much higher than expected, which can be attributed to the smaller sample size of this group of patients (n=98) combined with the patients' worse general condition and greater comorbidities at the time of diagnosis.

Although this is the only study that compares laparoscopic RC and LC with higher RC mortality in the literature, we believe that the result is attributable to the service's patient profile, with older age, greater comorbidities, higher rate of anemia, and consequently, need for pre-operative transfusion. Among studies of open surgery, only Benedix et al.^1^ observed higher mortality in RC, also justified by older age and preoperative comorbidity.

Some limitations of the present study must be considered. Due to the retrospective design, information bias cannot be forgotten, especially related to postoperative complications. However, this bias is expected and was present in both groups analyzed randomly.

There are still few studies comparing laparoscopic right and left colectomy in cancer, intraoperatively and postoperatively. The morbidity and mortality of both resections is acceptable, making this the approach of choice for colon cancer treatment to date.

## CONCLUSIONS

Surgical and oncological results in patients undergoing right and left laparoscopic colectomy are similar, making laparoscopy the preferred approach for both surgeries. However, the results should not be generalized and more studies should be done to confirm this finding.

Although right colectomy is the surgery of choice nowadays to begin the experience in laparoscopic colorectal surgery, its frequent anatomical variations, patient severity, and greater morbidity and mortality can modify this current scenario.
